# Prognostic Value of Lymphocyte-to-Monocyte Ratio (LMR) in Cancer Patients Undergoing Immune Checkpoint Inhibitors

**DOI:** 10.1155/2022/3610038

**Published:** 2022-12-23

**Authors:** Luying Wan, Chunlan Wu, Shuimei Luo, Xianhe Xie

**Affiliations:** ^1^Department of Oncology, Molecular Oncology Research Institute, The First Affiliated Hospital of Fujian Medical University, Fuzhou 350005, China; ^2^Department of Oncology, National Regional Medical Center, Binhai Campus of the First Affiliated Hospital, Fujian Medical University, Fuzhou 350212, China; ^3^Fujian Key Laboratory of Precision Medicine for Cancer, The First Affiliated Hospital, Fujian Medical University, Fuzhou 350005, China

## Abstract

**Background:**

There is accumulating evidence that the lymphocyte-to-monocyte ratio (LMR) is related to the outcomes of cancer patients treated with immune checkpoint inhibitors (ICIs). However, the results remain controversial.

**Method:**

Electronic databases were searched to retrieve the studies that explore the relationship between LMR and the efficacy of ICIs. The primary endpoints were overall survival (OS) and progression-free survival (PFS), evaluated by the hazard ratios (HRs) with 95% confidence intervals (CI), and the secondary endpoints included disease control rate (DCR) and immune-related adverse events (irAEs), assessed by the odd ratios (ORs) with 95% CI.

**Results:**

A total of 27 studies involving 4,322 patients were eligible for analysis. The results indicated that increased LMR at baseline was associated with a superior OS (HR: 0.46, 95% CI: 0.39-0.56, *p* < 0.001), PFS (HR: 0.60, 95% CI: 0.49-0.74, *p* < 0.001), and DCR (OR: 3.16, 95% CI: 1.70-5.87, *p* < 0.001). Posttreatment LMR was linked to a better PFS (HR: 0.46, 95% CI: 0.29-0.71, *p* = 0.001), but failed to show this correlation in the analysis of OS and DCR. No correlation existed between LMR and irAEs regardless of the testing time (baseline or posttreatment). Subgroup analyses focusing on baseline LMR revealed that higher baseline LMR possessed a better OS in renal cell cancer (RCC) arm, nonsmall cell lung cancer (NSCLC) arm, multiple cancer arm, monotherapy arm, LMR <2 arm, LMR ≥2 arm, western countries arm, eastern countries arm, and anti-PD-1 arm. Higher baseline LMR correlated with better PFS in RCC arm, NSCLC arm, gastric cancer (GC) arm, multiple cancer arm, LMR <2 arm, LMR ≥2 arm, western countries arm, and eastern countries arm.

**Conclusions:**

Higher LMR at baseline was positively correlated with a superior OS, PFS, and DCR for ICIs, but not with irAEs.

## 1. Introduction

Cancer immunotherapy has made great strides with the advancement of multiple forms of treatment, including immune checkpoint inhibitors (ICIs), oncolytic virus therapies, cancer vaccines, cytokine therapies, and adoptive cell transfer [1, 2]. Impressively, some incurable tumors with poor prognoses, such as metastatic melanoma and nonsmall cell lung cancer (NSCLC), have been recognized as sensitive to immunotherapy, and therefore have acquired a long-term maintenance of remission [3]. ICIs, which stimulate the host immune system to eliminate cancer cells by inhibiting the immune checkpoint pathway, are the most representative agents [4–6]. However, only a proportion of patients achieved a clinically desirable efficacy, and due to the high price and potential severe immune-related adverse events (irAEs) of ICIs, seeking for effective biomarkers to predict better respond to ICIs remains the current challenge in clinical practice [7–9].

Biomarkers identification is an important area in the diagnosis and management of malignant tumors. During the past decades, evidence-based meta-analyses have increased exponentially, which enrich our understanding of particular associations and trends in contemporary literature by improving statistical power and reducing outlier studies [10]. At the same time, there is a growing need to develop fast and easily accessible biospecimens, such as blood and urine, and corresponding biomarkers among clinical communities [11, 12]. So far, mismatch repair deficiency (MMR), programmed cell death-ligand 1 (PD-L1), tumor mutational burden (TMB), and gut microbiota (GM) features [8, 13–15] have been regarded as the best available biomarkers to predict the efficacy of ICIs, but they are confronted with some limitations, including high cost, obstacles in obtaining tissue samples, and lack of robust prognostic accuracy. Thus, there is an urgent need to identify novel biomarkers to precisely predict the therapeutic effects of ICIs.

Tumor associated inflammation is one of the hallmarks of cancer that enables tumorigenesis, angiogenesis, and tumor progression [16, 17]. Epidemiological researches have manifested that about a quarter of human cancers are associated with chronic inflammation [18]. Neutrophils involve in both innate and adaptive immune response and promote the tumor growth by secreting tumor growth factors that assist invasion and metastasis and promote angiogenesis [19, 20]. Monocytes participate in and prompt the process of inflammation by differentiating into either dendritic cells or tissue macrophages within tissue microenvironment [21]. T lymphocytes can recognize and kill tumor cells and correlate with a favorable clinical prognosis in several human tumors [22]. Thus, the blood-derived parameters, which can indicate systemic inflammatory responses, have proved to be related with the survival of cancer patients. Among these markers, neutrophil-lymphocyte ratio (NLR), lymphocyte-to-monocyte ratio (LMR), platelet-lymphocyte ratio (PLR), and systemic immune-inflammatory (SII) are intensively investigated, and a wealth of studies have demonstrated the significant association between these biomarkers and survival in malignant tumors. For instance, higher NLR and PLR, and lower LMR indicate a poor prognosis in lung cancer, colorectal cancer, renal cell carcinoma, melanoma, and so on [23–29]. In addition, blood-derived parameters can be easily utilized in routine work. Therefore, study on whether there is association between peripheral blood biomarkers and clinical outcomes of ICIs is on the agenda.

Recently, several meta-analyses have been published focusing on the relationship between NLR or PLR and the efficacy of ICIs, but to our knowledge, only one on LMR, which recruited limited four studies in nonsmall cell lung cancer with endpoints of only overall survival (OS) and progression-free survival (PFS) [30]. Since previous studies yielded controversial conclusions regarding the association between LMR and the efficacy of ICIs, we conducted an updated and comprehensive investigation which recruited 27 studies reporting the endpoints of OS, PFS, disease control rate (DCR), and irAEs and performed detailed subgroup analyses based on the testing time of LMR (baseline or posttreatment), cancer types, combination medication, LMR cut-off, study region, and types of ICIs.

## 2. Materials and Methods

### 2.1. Search Strategy

This meta-analysis was designed and conducted according to the Preferred Reporting Items for Systematic Reviews and Meta-Analysis (PRISMA) checklist. PubMed, Cochrane Library, and EMBASE were searched for eligible studies up to September 4, 2022. The search strategy based on the following key words: “immune checkpoint inhibitor”, “ICIs”, “immune checkpoint blocker”, “PD-L1 inhibitor”, “PD 1 inhibitor”, “programmed cell death protein 1 inhibitor”, “programmed death ligand 1 inhibitor”, “cytotoxic T lymphocyte associated protein 4 inhibitor”, “CTLA-4 inhibitor”, “pembrolizumab”, “nivolumab”, “tremelimumab”, “avelumab”, “toripalimab”, “envafolimab”, “sintilimab”, “camrelizumab”, “cemiplimab”, “tislelizumab”, “cetrelimab”, “pidilizumab”, “triprizumab”, “atezolizumab”, “durvalumab”, “ipilimumab”, “monocyte”, “lymphocyte”, “monocyte to lymphocyte ratio (MLR)”, and “lymphocyte to monocyte ratio (LMR)”, and articles were limited to English-language publications. If the title and abstract failed to provide enough information, a full text evaluation was conducted. In addition, the references list of all related articles were manually reviewed to identify potential relevant studies. Reviews, meta-analysis, case reports, comments, and conference abstracts without original data were excluded.

### 2.2. Study Selection

Two independent investigators individually screened the titles and abstracts, and full-text articles were obtained and evaluated to acquire eligible researches. Inclusion criteria were as follows: (1) patients were pathologically diagnosed as solid malignant tumors; (2) ICI agents were administered alone or in combination; (3) therapeutic outcomes (OS, PFS, and DCR) were determined by RECIST criteria, or the association between LMR and irAEs were evaluated; (4) a hazard ratio (HR) and/or an odds ratio (OR) with 95% confidence interval (CI) could be extracted or calculated from the literature; (5) patients were assigned into high or low LMR groups by cutoff value; and (6) articles were published in full texts.

### 2.3. Data Extraction

The Newcastle-Ottawa Quality Assessment Scale (NOS) was adopted to evaluate the quality of researches, and those scoring five or more stars were considered of medium to high quality. Studies were screened and evaluated by two independent investigators according to inclusion criteria. Any disagreement were settled by consultation. Data extracted were the first author's name, publication year, country, study type, tumor type, sample size, line of therapy, type of ICIs, combined medication, the testing time, and cut-off of LMR, age, HRs with 95% CI of OS and PFS, ORs with 95% CI of DCR and irAEs.

### 2.4. Statistical Analysis

The primary endpoints were OS and PFS and the secondary endpoints were DCR and irAEs. The pooled HRs/ORs with 95% CI were evaluated to identify the association between LMR and the efficacy or adverse events of ICIs. Results relating to MLR was converted into the form of LMR. The median value of LMR was used as the cut-off value. Statistical analyses were conducted with Stata version 12. Heterogeneity among recruited studies was checked by *I*^2^ tests: *I*^2^ > 50% or *P* < 0.1 means substantial heterogeneity and a random-effects model was used; otherwise, a fixed-effects model was applied. A statistically significant difference was set as *p* <0.05. Funnel plot and Egger's test were performed to assess the publication bias. Sensitivity analyses were carried out by excluding one article each time to verify the reliability of our results.

## 3. Result

### 3.1. Study Characteristics

**Figure 1:**
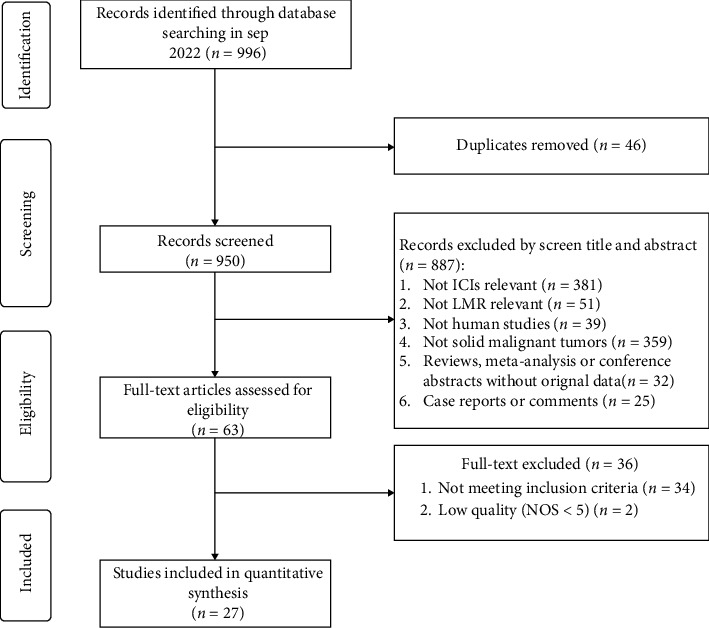
Flowchart of study selection procedure.

A total of 996 articles were retrieved from the PubMed, EMBASE, and Cochrane Library. After removing duplicates, 950 studies were left. By examining titles and abstracts, 887 were excluded due to non-ICIs or non-LMR studies, nonhuman studies, nonmalignant tumors, reviews, comments, case reports, meta-analyses, and conference abstracts without original data; consequently, 63 articles were identified for further study. Through full-text review of these literature, 36 were disregarded due to not meeting the inclusion criteria or low quality (NOS < 5), and 27 studies incorporating 4,322 patients were finally identified as eligible for this meta-analysis (Figure 1).

All studies were retrospective and were published between 2017 and 2022. Of these studies, 11 were conducted in China, 10 in Japan, 2 in Italy, 2 in USA, 1 in Korea, and 1 in Spain. 7 on NSCLC, 3 on gastric cancer (GC), 2 on hepatocellular carcinoma (HCC), 3 on renal cell cancer (RCC), 1 on small cell lung cancer (SCC), 1 on melanoma, 1 on esophageal cancer (EC), 1 on biliary tract cancer (BTC), 2 on lung cancer (LC), 1 on urothelial carcinoma (UC), and 5 on two or more types of solid tumors. Meanwhile, all the patients treated with ICIs: anti-PD-1/PD-L1 (Pembrolizumab, Nivolumab, Atezolizumab, Sintilimab, Camrelizumab, Triprizumab, and Toripalimab) or anti-CTLA-4; 24 studies measured the LMR at baseline and 5 studies measured LMR after treatment, with 2 evaluated LMR at both baseline and posttreatment; 21 trails had OS, 15 trails PFS, 8 trails DCR, and 6 trails irAEs. Characteristics of these studies enrolled are listed in Table 1.

### 3.2. Quality Assessment

All the included 27 studies were rated as moderate or high quality with a score from five to eight based on the NOS criteria, which were eligible for meta-analysis (Table 1).

### 3.3. Main Results

#### 3.3.1. LMR and OS

**Figure 2:**
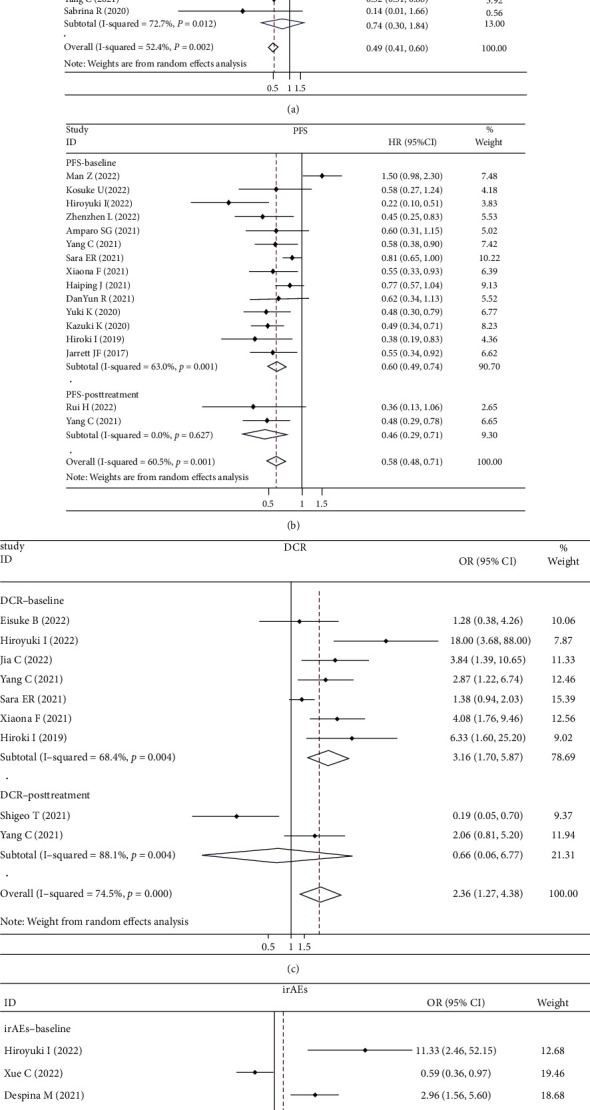
Forest plots for (a) overall survival (OS), (b) progression-free survival (PFS), (c) disease control rate (DCR), and (d) immune-related adverse event (irAEs).

Twenty-one cohorts incorporating 2,739 individuals were included in our analysis of the association between LMR and OS, with 17 cohorts provided only baseline LMR values, 2 only posttreatment LMR values, and 2 both baseline and posttreatment LMR values. Polled analysis showed high LMR value was significantly associated with a better OS in cancer patients treated with ICIs (HR: 0.49, 95% CI: 0.41-0.60, *p* < 0.001, Figure 2(a)), but with an obvious heterogeneity (*I*^2^ = 52.4%, *p* = 0.002). Hence, a further analysis was performed according to the testing time of LMR. Results showed that high baseline LMR contributed to a better OS (HR: 0.46, 95% CI: 0.39-0.56, *p* < 0.001, Figure 2(a)) with significant heterogeneity (*I*^2^ = 42.5%, *p* = 0.027), but there was no relationship between posttreatment LMR and OS (HR: 0.74, 95% CI: 0.30-1.84, *p* = 0.51, Figure 2(a)).

**Figure 3:**
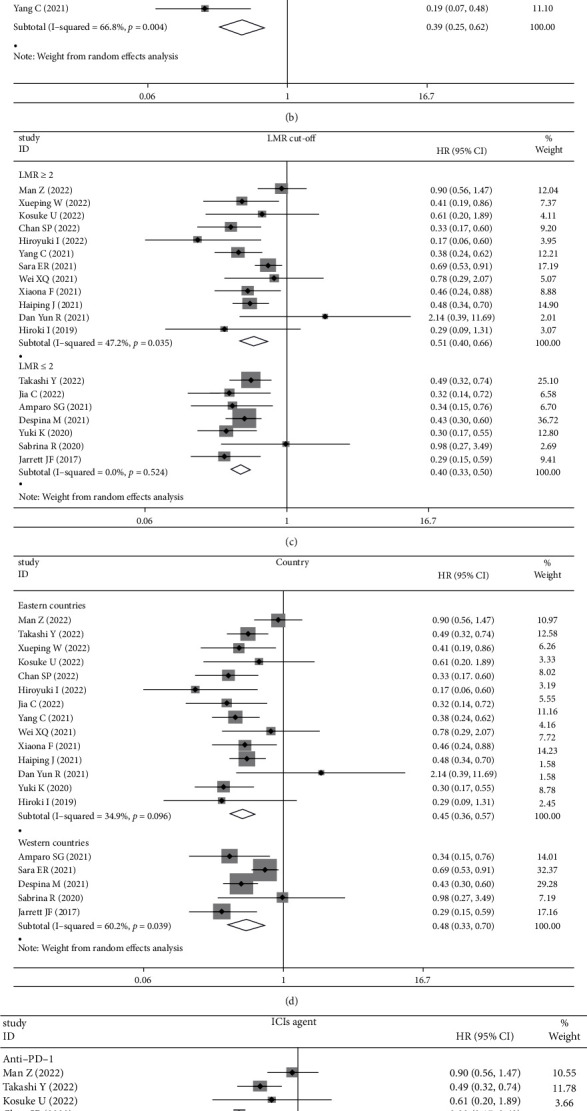
(a) The pooled HRs for overall survival (OS) by LMR at baseline stratified on tumor types (RCC, NSCLC, GC, and multiple); (b) whether monotherapy or combined therapy; (c) LMR cut-off (<2 and ≥2); (d) countries (western countries and eastern countries); (e) type of ICI agents (anti-PD-1 and anti-PD-L1); (f) funnel plot for the evaluation of publication bias considering the association between the LMR at baseline and OS (19 studies).

Therefore, subgroup analyses focused only on baseline LMR, and high baseline LMR indicated a better OS in RCC arm (HR: 0.66, 95% CI: 0.51-0.86, *p* = 0.002, Figure 3(a)), NSCLC arm (HR: 0.35, 95% CI: 0.24-0.52, *p* < 0.001, Figure 3(a)), multiple cancer arm (HR: 0.45, 95% CI: 0.36-0.57, *p* < 0.001, Figure 3(a)), monotherapy arm (HR: 0.39, 95% CI: 0.25-0.62, *p* < 0.001, Figure 3(b)), LMR ≥2 arm (HR: 0.51, 95% CI: 0.40-0.66, *p* < 0.001, Figure 3(c)), LMR <2 arm (HR: 0.40, 95% CI: 0.33-0.50, *p* < 0.001, Figure 3(c)), eastern countries arm (HR: 0.45, 95% CI: 0.36-0.57, *p* < 0.001, Figure 3(d)), western countries arm (HR: 0.48, 95% CI: 0.33-0.70, *p* < 0.001, Figure 3(d)), and anti-PD-1 arm (HR: 0.49, 95% CI: 0.38-0.62, *p* < 0.001, Figure 3(e)). However, higher baseline LMR values indicated a better OS in GC group (HR: 0.74, 95% CI: 0.14-3.83, *p* = 0.718, Figure 3(a)), combination therapy group (HR: 0.70, 95% CI: 0.45-1.10, *p* = 0.12, Figure 3(b)), and anti-PD-L1 group (HR: 0.44, 95% CI: 0.18-1.12, *p* = 0.085, Figure 3(e)) without statistical significance.

#### 3.3.2. LMR and PFS

Fifteen cohorts provided the data of LMR and PFS, in which 13 cohorts displayed baseline LMR values, 1 cohort posttreatment LMR values, and 1cohort both baseline and posttreatment LMR values. As with the results of OS analyses, a higher LMR was also associated with a better PFS in cancer patients treated with ICIs (HR: 0.58, 95% CI: 0.48-0.71, *p* < 0.001, Figure 2(b)) in both baseline and posttreatment LMR studies (HR: 0.60, 95% CI: 0.49-0.74, *p* < 0.001; HR: 0.46, 95% CI: 0.29-0.71, *p* = 0.001, respectively, Figure 2(b)), but with an obvious heterogeneity (*I*^2^ = 60.5%, *p* = 0.001).

**Figure 4:**
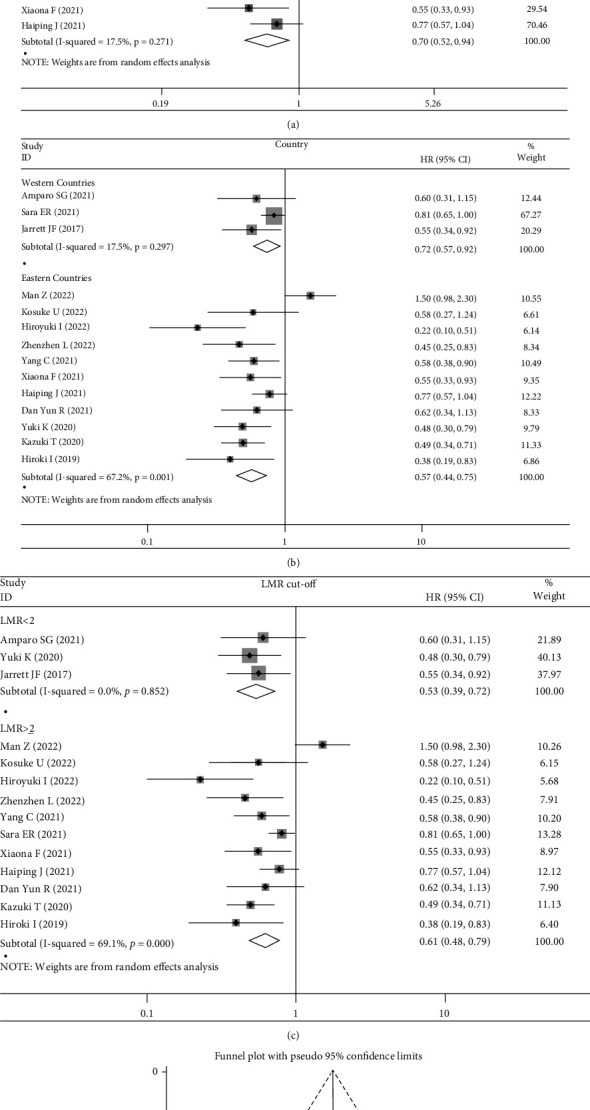
(a) The pooled HRs for progression-free survival (PFS) by LMR at baseline stratified on tumor types (RCC, NSCLC, GC, and multiple); (b) LMR cut-off (<2 and ≥2); (c) and countries (eastern countries and western countries); (d) funnel plot for the evaluation of publication bias considering the association between the LMR at baseline and PFS (14 studies).

Because only 2 researches provided the data of posttreatment LMR, which were insufficient for subgroup analysis, and further stratified analyses were performed based on the baseline LMR studies. Results exhibited that high baseline LMR led to a better PFS in RCC arm (HR: 0.63, 95% CI: 0.40-0.99, *p* = 0.047, Figure 4(a)), NSCLC arm (HR: 0.50, 95% CI: 0.39-0.66, *p* < 0.001, Figure 4(a)), GC arm (HR: 0.59, 95% CI: 0.42-0.84, *p* = 0.003, Figure 4(a)), multiple cancer arm (HR: 0.70, 95% CI: 0.52-0.94, *p* = 0.019, Figure 4(a)), western countries arm (HR: 0.72, 95% CI: 0.57-0.92, p =0.008, Figure 4(b)), eastern countries arm (HR: 0.57, 95% CI: 0.44-0.75, *p* < 0.001, Figure 4(b)), LMR <2 arm (HR: 0.53, 95% CI: 0.39-0.72, *p* < 0.001, Figure 4(c)), and LMR ≥2 arm (HR: 0.61, 95% CI: 0.48-0.79, *p* < 0.001, Figure 4(c)).

#### 3.3.3. LMR and DCR

Eight cohorts incorporating 1,117 cases provided the data of LMR and DCR, with 1 cohort displayed both baseline and posttreatment LMR, 6 baseline LMR, and 1 posttreatment LMR. Similarly, a higher LMR value was correlated with a better DCR (OR: 2.36, 95% CI: 1.27-4.38, *p* = 0.006, Figure 2(c)), but with significant heterogeneity (*I*^2^ = 74.5%, *p* < 0.001). Subgroup analysis were then conducted according to the testing time of LMR, which displayed a positive association between higher LMR at baseline and a better DCR (OR: 3.16, 95% CI: 1.70-5.87, *p* < 0.001, Figure 2(c)) but no obvious correlation between LMR at posttreatment and DCR (OR: 0.66, 95% CI: 0.06-6.77, *p* = 0.724, Figure 2(c)).

#### 3.3.4. LMR and irAEs

Six studies with 1,852 patients were available in the analysis of the association between LMR and irAEs of any grade, with 5 displayed baseline LMR and 1 posttreatment LMR. Our pooled analysis showed that LMR did not exist a correlation with irAEs regardless of the testing time (OR: 1.26, 95% CI: 0.53-3.02, *p* = 0.599, Figure 2(d)).

### 3.4. Publication Bias

Among the above results, the analysis of the relationships of LMR at baseline with OS and PFS included enough articles (>10 studies) and funnel plot (Figure 3(f)) and Egger's test were conducted. The shape of the funnel plot suggested no publication bias for recruited studies on OS (Egger: *p* = 0.33) (Figure 3(f)), while there was a publication bias for PFS (Egger: *p* = 0.03) (Figure 4(d)). Meanwhile, because of the limited number of studies for meaningful assessment (<10 studies), the publication bias was not performed in other analyses.

### 3.5. The Sensitivity Analysis

**Figure 5:**
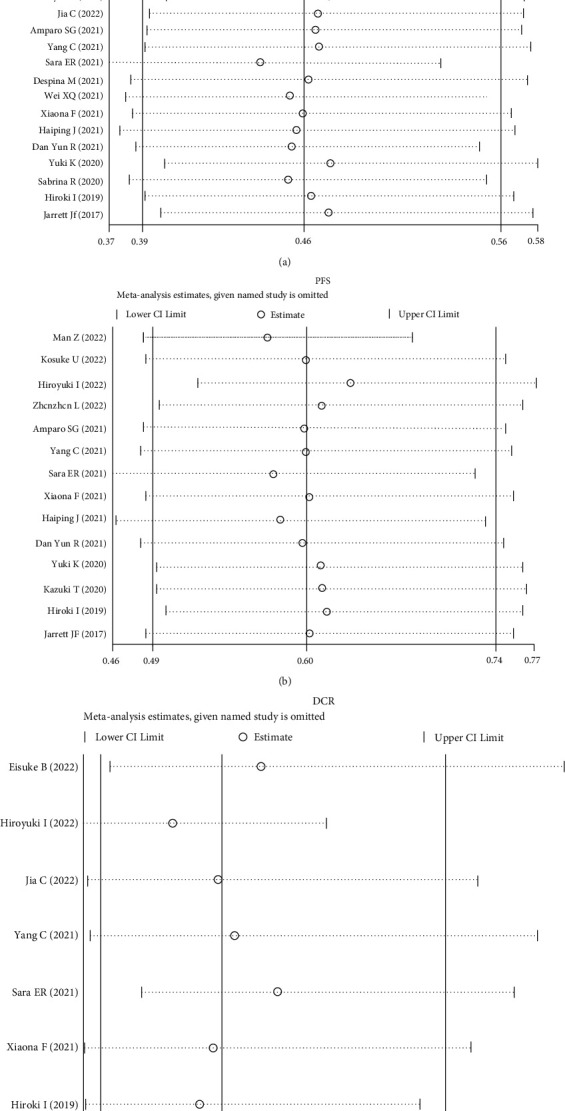
Sensitivity analysis of (a) overall survival (OS), (b) progression-free survival (PFS), and (c) disease control rate (DCR).

We performed sensitivity analysis for baseline LMR due to their clinical significance by excluding one single study from the primary analysis, which proved that no individual study influenced the results on OS, PFS, and DCR, suggesting the results were relatively credible (Figure 5).

## 4. Discussion

The relationship between inflammation and neoplasm progression or metastasis has long been discussed. Blood-derived parameters, which are easily accessible and reproducible indicators of systemic inflammation, have already been used as objective biomarkers for predicting the prognoses of cancer patients [31, 32]. In light of this, increasing studies have explored whether some of them possess the ability to predict the efficacy of immunotherapy. However, among these markers, LMR is relatively less investigated. LMR was initially identified in hematological malignancies as a prognostic predictor, then a growing body of work demonstrated its positive association with better prognoses in many solid tumors, including lung cancer, gastric cancer, breast cancer, and melanoma [24–26, 33, 34]. For example, in patients of melanoma treated with ipilimumab, higher level of monocyte was found in cases that did not respond to this agent [35]. Similarly, higher baseline absolute lymphocyte count indicated an improved OS in patients treated with pembrolizumab [36]. In this meta-analysis, we investigated the association between LMR and the therapeutic effect of ICIs based on 27 studies incorporating 4,322 patients and multiple tumor types, and the results displayed that higher baseline LMR was positively correlated with a superior OS, PFS, and DCR for ICIs, indicating that higher LMR may be a signal for better efficacy for patients receiving ICIs treatment.

LMR, which is calculated by lymphocytes and monocytes, represents the antitumor immunity and tumor burden in human body [37]. On the one hand, tumor-infiltrating lymphocytes (TILs) are transformed from circulating lymphocytes in tumor microenvironments and well-known to contribute to antitumor immunity through their cytolytic activity. Therefore, insufficient numbers of lymphocyte are regarded as a contributing factor to the under-activation of the immunologic reaction to the tumor [38], which indicates poor clinical outcomes in multiple cancer types [39]. Previous studies showed that higher level of tumor infiltrating CD8^+^ T cell predicted better efficacy of ICIs in melanoma and clear cell renal cell carcinoma patients [40, 41]. In addition, B lymphocytes are also reported to be associated with good clinical response in cancer patients receiving anti–PD-1 therapy [42, 43]. On the other hand, monocytes infiltrate tumors and evolved into tumor-associated macrophages (TAMs) in response to chemokines, which involved in tumor proliferation, invasion, metastasis, and angiogenesis [44–46]. In gastric cancer, TAMs have been reported to suppress the function of cytotoxic T cells through the PD-1/PD-L1 pathway [47] and indicate poor prognoses [48, 49]. Consistently, in in vivo experiment, TAMs can lead to resistance of PD-1 inhibitors [50]. Therefore, LMR was thought to reflect host immune status and have the potential to serve as a predictor of therapeutic effect of ICIs treatment.

The differential responses to ICIs can be linked to the diversity of individual innate immune system and some other factors [7, 8], including the patient's specific GM. As accumulating evidence has demonstrated the critical role of GM in modulating the host's immune system [8, 51], GM manipulation is further proved to be a powerful therapeutic strategy to affect ICIs efficacy and irAEs [52, 53]. For instance, antibiotic administration could lead to a disrupted GM and therefore compromise the therapeutic effect of ICIs, while fecal microbiota transplantation (FMT) resulted in overcoming of anti-PD-1 therapy [53–55]. In addition, previous study shed light on the role of neutrophil-lymphocyte ratio (NLR) as a systemic inflammation marker to reflect the status of GM, and individuals with lower NLR showed increased diversity in their gut microbiota [56]. In turn, short-chain fatty acids (SCFAs), one of metabolites produced by microbes from components in the gut, can promote both the effector and regulatory effects of T cells and the antibody production, and may therefore enhance the host's immunity [57]. Taken together, these results revealed the interaction of microbiota, hematological inflammatory indicators, and ICIs efficacy, through which the inflammatory markers may predict the clinical outcomes of ICIs therapy.

This study displayed that baseline LMR is positively correlated with OS, PFS, and DCR, while posttreatment LMR failed to show this correlation in the analysis of OS and DCR. This may be ascribed to the limited number of studies reporting the results of posttreatment LMR and the inconsistencies of the testing time of LMR, which varies from 2 weeks after initial administration to 8 weeks. Previous research indicated that the least time of activated leukocytes “truly” mobilize into peripheral blood is 4 weeks [58], which may partly explain the discordant conclusion of articles reporting LMR at posttreatment. Hence, future studies may more specifically investigate whether the different testing time of posttreatment LMR could influence the outcomes and whether changes of LMR between pre- and post-ICIs correlate with the clinical efficacies.

At present, the precise mechanisms of the presentation of irAEs have not been fully elucidated. One explanation is that tumor cells and the affected tissue have shared antigens, and activated CD8-positive T-lymphocytes cannot distinguish between them and attack normal tissue cells unexpectedly [59]. Other potential mechanisms include subclinical autoimmune responses and microbiome [60, 61]. A number of studies have examined the relationships of peripheral blood biomarkers with the risk of irAEs, which yielded different conclusions [62, 63]. In this study, we observed that LMR had no relationship with irAEs both before or post ICIs treatment, but in consideration of the limited data, prospective studies with larger patient cohorts and more detailed patients' clinical information are needed.

This study encountered several limitations: first, all the recruited studies were retrospective, while no randomized controlled trial (RCT) was available, which may lead to potential confounders. Second, subgroup analysis based on specific ICIs agent were not able to conduct due to the less comprehensive data. Third, there was publication bias for pooled PFS in the analysis of LMR at baseline. Nonetheless, our results are still interesting because few meta-analyses focus on the relationship between LMR and the clinical outcomes of ICIs.

In conclusion, this study showed that the value of baseline LMR is positively associated with a better OS, PFS, and DCR in cancer patients undergoing ICI therapy, and subgroup analyses on tumor types, ICIs agents, combination therapy, cutoff value of LMR, and study regions exhibited similar results or trends, indicating the promising prognostic value of LMR on ICIs therapy in clinical practice.

## Figures and Tables

**(a):** 

Author	Year	Country	Study type	Cancer type	Sample (H/L)	Male	ICIs agents	Line of therapy	Combined medication	Testing time	LMR cut-off	Outcome	Age	Quality evaluation
Man [64]	2022	China	R	HCC	160 (61/99)	129	Pembro/Nivo/+Lenva; Sinti/Camre +lenva/Sora	1st-line	Combined therapy	Baseline	3.6	OS, PFS	58 (26-86)	7
Takashi [65]	2022	Japan	R	UC	149	126	Pembro	2nd-line or later	NA	Baseline	1.7	OS	72 (67–77)	6
Eisuke [66]	2022	Japan	R	Multiple	61 (32/29)	49	Pembro/Nivo/Nivo followed by Pembro	2nd-line or later	Monotherapy, combined therapy	Baseline	1.8 or 2.6	DCR	71 (46-86)	6
Xueping [67]	2022	China	R	LC	125 (69/56)	90	Nivo, Pembro, Atezo	NA	Monotherapy, combined therapy	Baseline	2.3	OS	55	6
Kosuke [68]	2022	Japan	R	RCC	38 (25/13)	30	Nivo	2nd-line or later	Monotherapy	Baseline	3.3	OS, PFS	68 (44–78)	5
Chan [69]	2022	Korea	R	BTC	68(29/39)	NA	Pembro	2nd-line or later	Monotherapy	Baseline	2.5	OS	66	6
Hiroyuki [70]	2022	Japan	R	EC	41(20/21)	34	Nivo	2nd-line or later	Monotherapy	Baseline	2.2	OS, PFS, DCR, irAEs	68 (51-81)	6
Rui [31]	2022	China	R	HCC	110 (79/31)	100	Anti-PD-1	NA	Combined therapy	Posttreatment	1.8	OS, PFS	55 (31-84)	6
Xue [71]	2022	China	R	Multiple	1,047	713	Anti-PD-(L)1	1st-line or later	Monotherapy, combined therapy	Baseline	3.4	irAEs	60	6
Jia [72]	2022	China	R	NSCLC	85(63/22)	62	Pembro/Nivo/Sinti/Toripa +/- chemo +/-antiangiogenesis	1st-line or later	Monotherapy, combined therapy	Baseline	1.8	OS, DCR	66 (47-80)	7
Zhenzhen [73]	2022	China	R	NSCLC, SCLC	66	47	Anti-PD-(L)1	1st-line or later	NA	Baseline	2.1	PFS	NA	5
Amparo [74]	2021	Spain	R	NSCLC	51(27/24)	37	Pembro	1st-line	Monotherapy	Baseline	1.9	OS, PFS	66 (46–85)	8
Shigeo [75]	2021	Japan	R	GC	51(25/26)	NA	Nivo	2nd-line or later	Monotherapy	Posttreatment	3.3	OS, DCR	69 (40–84)	8
Yang [76]	2021	China	R	GC	139 (71/68)	103	Anti-PD-(L)1+ chemo/anti-VEGF/anti-HER/anti-CTLA-4	1st-line or later	Monotherapy, combined therapy	Baseline, posttreatment	3.5	OS, PFS, DCR	60 (51–67)	7
Saeka [77]	2021	Japan	R	NSCLC	171	113	Nivo	2nd-line or later	Monotherapy	Posttreatment	2.2	irAEs	64 (56–69)	6
Sara [78]	2021	Italy	R	RCC	571	402	Nivo	2nd-line or late	Monotherapy	Baseline	2.6	OS, PFS, DCR	61 (49–73)	8
Despina [79]	2021	USA	R	Multiple	390	275	Anti-PD-(L)1, anti-CTLA-4 or combination	NA	Monotherapy, combined therapy	Baseline	1.4	OS, irAEs	65	6
Wei [80]	2021	China	R	SCLC	53	34	Atezo + chemo +/- trilaciclib	1st-line	Combined therapy	Baseline	2.7	OS	NA	6
Saeka [81]	2021	Japan	R	NSCLC	92	64	Pembro	1st-line or later	Monotherapy	Baseline	1.5	irAEs	60 (34–85)	6
Xiaona [82]	2021	China	R	Multiple	111 (61/50)	56	Anti-PD-1	1st-line or late	NA	Baseline	3.2	OS, PFS, DCR, irAEs	NA	7
Haiping [83]	2021	China	R	Multiple	207	156	Sinti+/- chemo	1st-, 2nd-, or 3rd-line	Monotherapy, combined therapy	Baseline	2.8	OS, PFS	56 (24-72)	9
DanYun [84]	2021	China	R	GC	53(21/32)	NA	Toripa	2nd-line or later	Monotherapy	Baseline	2.8	OS, PFS	60 (52–66)	6
Yuki [85]	2020	Japan	R	NSCLC	81	37	Atezo	2nd-line or later	Monotherapy	Baseline	1.5	OS, PFS	71 (42–84)	7
Sabrina [86]	2020	Italy	R	NSCLC	65(16/49)	44	Nivo	2nd-line or later	NA	Baseline, posttreatment	1.4	OS	68 (39-86)	6
Kazuki [87]	2020	Japan	R	NSCLC	146	NA	Nivo, Pembro	1st-line or later	NA	Baseline	2.1	PFS	NA	6
Hiroki [88]	2019	Japan	R	RCC	58(21/37)	45	Nivo	2nd-line or later	NA	Baseline	3.3	OS, PFS, DCR	34	6
Jarrett [89]	2017	USA	R	Melanoma	133 (98/35)	87	Pembro	NA	NA	Baseline	1.7	OS, PFS	61	8

**(b):** 

Author	Year	Baseline				Posttreatment					
		HR for OS and 95% CI	HR for PFS and 95% CI	OR for DCR and 95% CI	OR for irAEs and 95% CI	HR for OS and 95% CI	HR for PFS and 95% CI	OR for DCR and 95% CI	OR for irAEs and 95% CI		
Man	2022	0.90 (0.56-1.47)	1.50 (0.98-2.30)	NA	NA	NA	NA	NA	NA		
Takashi	2022	0.49 (0.32-0.74)	NA	NA	NA	NA	NA	NA	NA		
Eisuke	2022	NA	NA	1.28 (0.38-4.26)	NA	NA	NA	NA	NA		
Xueping	2022	0.41(0.19-0.86)	NA	NA	NA	NA	NA	NA	NA		
Kosuke	2022	0.61 (0.20-1.89)	0.58 (0.27-1.24)	NA	NA	NA	NA	NA	NA		
Chan	2022	0.33 (0.17-0.60)	NA	NA	NA	NA	NA	NA	NA		
Hiroyuki	2022	0.17 (0.06-0.60)	0.22 (0.10-0.51)	18.00 (3.68-88.00)	11.33 (2.46-52.15)	NA	NA	NA	NA
Rui	2022	NA	NA	NA	NA	0.61 (0.21-1.79)	0.36 (0.13-1.06)	NA	NA		
Xue	2022	NA	NA	NA	0.59 (0.36–0.97)	NA	NA	NA	NA	
Jia	2022	0.32 (0.14-0.72)	NA	3.84 (1.39-10.65)	NA	NA	NA	NA	NA		
Zhenzhen	2022	NA	0.45 (0.25-0.83)	NA	NA	NA	NA	NA	NA		
Amparo	2021	0.34 (0.15-0.76)	0.60 (0.31-1.15)	NA	NA	NA	NA	NA	NA		
Shigeo	2021	NA	NA	NA	NA	2.17 (0.99-4.63)	NA	0.19 (0.05-0.70)	NA		
Yang	2021	0.38(0.24-0.62)	0.58 (0.38-0.90)	2.87 (1.22-6.74)	NA	0.52 (0.31-0.88)	0.48 (0.29-0.78)	2.06 (0.81-5.20)	NA		
Saeka	2021	NA	NA	NA	NA	NA	NA	NA	1.79 (0.90-3.56)		
Sara	2021	0.69 (0.53-0.91)	0.81 (0.65-1.00)	1.38 (0.94-2.03)	NA	NA	NA	NA	NA		
Despina	2021	0.43 (0.30-0.60)	NA	NA	2.96 (1.56-5.60)	NA	NA	NA	NA		
Wei	2021	0.78 (0.29-2.07)	NA	NA	NA	NA	NA	NA	NA		
Saeka	2021	NA	NA	NA	0.12 (0.03-0.52)	NA	NA	NA	NA		
Xiaona	2021	0.46 (0.24-0.88)	0.55 (0.33-0.93)	4.08 (1.76-9.46)	1.01 (0.44-2.34)	NA	NA	NA	NA		
Haiping	2021	0.48 (0.34-0.70)	0.77 (0.57-1.04)	NA	NA	NA	NA	NA	NA		
DanYun	2021	2.14 (0.39-11.69)	0.62 (0.34-1.13)	NA	NA	NA	NA	NA	NA		
Yuki	2020	0.30 (0.17-0.55)	0.48 (0.30-0.79)	NA	NA	NA	NA	NA	NA		
Sabrina	2020	0.98 (0.27-3.49)	NA	NA	NA	0.14 (0.01-1.66)	NA	NA	NA		
Kazuki	2020	NA	0.49 (0.34-0.71)	NA	NA	NA	NA	NA	NA		
Hiroki	2019	0.29 (0.09-1.31)	0.38 (0.19-0.83)	6.33 (1.60-25.20)	NA	NA	NA	NA	NA		
Jarrett	2017	0.29 (0.15-0.59)	0.55 (0.34-0.92)	NA	NA	NA	NA	NA	NA		

NA, not available; R, retrospective; NSCLC, non-small cell lung cancer; GC, gastric cancer; RCC, renal cell carcinoma; SCC, small cell lung cancer; UC, urothelial carcinoma; LC, lung cancer; BTC, biliary tract cancer; EC, esophageal cancer; HCC, hepatocellular carcinoma; CRC, colorectal cancer; PLC, primary liver cancer; H/L, LMR high group/ low group; chemo, chemotherapy; ICIs, immune checkpoint inhibitors; Pembro, Pembrolizumab; Nivo, Nivolumab; Atezo, Atezolizumab; Sinti, Sintilimab; Camre, Camrelizumab; Tripri, Triprizumab; Toripa, Toripalimab; Lenva, lenvatinib; Sora, Sorafenib; Apa, apatinib; PD-(L)1, programmed death- (ligands) 1; CTLA-4, cytotoxic T lymphocyte antigen 4; OS, overall survival; PFS, progression free survival; ORR, objective response rate; DCR, disease control rate; irAEs, immune-related adverse events.

## Data Availability

Data extracted from included studies and used for all analyses are available in this published article.
